# Computational identification of deleterious synonymous variants in human genomes using a feature-based approach

**DOI:** 10.1186/s12920-018-0455-6

**Published:** 2019-01-31

**Authors:** Fang Shi, Yao Yao, Yannan Bin, Chun-Hou Zheng, Junfeng Xia

**Affiliations:** 1 College of Electrical Engineering and Automation, Anhui University, Hefei, 230601 Anhui China; 2 Institute of Physical Science and Information Technology, School of Computer Science and Technology, Anhui University, 111 Jiulong Avenue, Hefei, 230601 China

**Keywords:** Synonymous variant, Pathogenicity prediction, Feature selection, Random forest

## Abstract

**Background:**

Although synonymous single nucleotide variants (sSNVs) do not alter the protein sequences, they have been shown to play an important role in human disease. Distinguishing pathogenic sSNVs from neutral ones is challenging because pathogenic sSNVs tend to have low prevalence. Although many methods have been developed for predicting the functional impact of single nucleotide variants, only a few have been specifically designed for identifying pathogenic sSNVs.

**Results:**

In this work, we describe a computational model, IDSV (Identification of Deleterious Synonymous Variants), which uses random forest (RF) to detect deleterious sSNVs in human genomes. We systematically investigate a total of 74 multifaceted features across seven categories: splicing, conservation, codon usage, sequence, pre-mRNA folding energy, translation efficiency, and function regions annotation features. Then, to remove redundant and irrelevant features and improve the prediction performance, feature selection is employed using the sequential backward selection method. Based on the optimized 10 features, a RF classifier is developed to identify deleterious sSNVs. The results on benchmark datasets show that IDSV outperforms other state-of-the-art methods in identifying sSNVs that are pathogenic.

**Conclusions:**

We have developed an efficient feature-based prediction approach (IDSV) for deleterious sSNVs by using a wide variety of features. Among all the features, a compact and useful feature subset that has an important implication for identifying deleterious sSNVs is identified. Our results indicate that besides splicing and conservation features, a new translation efficiency feature is also an informative feature for identifying deleterious sSNVs. While the function regions annotation and sequence features are weakly informative, they may have the ability to discriminate deleterious sSNVs from benign ones when combined with other features. The data and source code are available on website http://bioinfo.ahu.edu.cn:8080/IDSV.

**Electronic supplementary material:**

The online version of this article (10.1186/s12920-018-0455-6) contains supplementary material, which is available to authorized users.

## Background

Synonymous single nucleotide variants (sSNVs), which do not alter protein sequences, were once thought to be functionally irrelevant. However, recent studies have shown that sSNVs are linked to human diseases [1–5]. For example, over 400 human diseases have been associated with sSNVs [6]. Studies analyzing the consequences of sSNVs have revealed that they play important roles in multiple biological processes, including transcription factor regulation [1], splicing regulation [7], microRNA binding, mRNA folding [8], and protein synthesis [9].

Although many bioinformatics methods have been developed for prioritizing single nucleotide variants, there are only a few methods available for sSNVs prediction. SilVA [10], the first bioinformatics method designed to discriminate between functional and non-functional sSNVs, is based on a random forest (RF) model trained with a diverse set of 26 features including conservation, codon usage, sequence features (CpG and relative mRNA position), exon splice enhancer/suppressor motifs, splicing site motifs, and mRNA folding. But only 33 deleterious SNVs were used for training the model in SilVA, which may limit its performance. Livingstone et al. built a support vector machine (SVM) model called DDIG-SN [11], which used six features to train and evaluate on nearly 900 disease-causing variants to discriminate disease-causing synonymous mutations. Their results suggest that the splicing feature is the dominant factor for disease-causing sSNVs. Zhang et al. developed another tool (termed regSNPs-splicing [12]) to prioritize sSNVs based on their impact on mRNA splicing and protein function. Recently, Gelfman et al. presented Transcript-inferred Pathogenicity (TraP) score [13], which can be used to evaluate a sSNV’s ability to cause disease by damaging a gene’s transcripts and protein products. Besides these tools specifically designed to predict functional sSNVs, several general-purpose variant effect predictors also implicated cover effects of sSNVs. For example, FATHMM-MKL [14] is an integrative approach to predict the functional consequences of both non-coding and coding sequence variants using various genomic annotations. CADD [15] is another general framework for predicting all possible types of human genetic variants based on SVM with a variety of features including scores calculated with other bioinformatics methods. Because both FATHMM-MKL and CADD are designed for predicting all types of pathogenic variants, it is not easy to assess the relative importance of various features devoted exclusively to sSNVs. In addition, several splicing-specific predictors can also be used to evaluate the harmfulness of sSNVs, including SPANR [16], a tool for evaluating how SNVs cause splicing mis-regulation, and MutPred Splice [17], a machine-learning approach for the identification of coding region substitutions that disrupt pre-mRNA splicing.

Although current computational methods achieve relative success for identifying deleterious sSNVs, they are still in the initial stage. Up to now, the biological properties that are responsible for deleterious sSNVs have not been fully understood. Consequently, the features previously identified as being correlated with deleterious sSNVs are still insufficient. In this paper, we developed a feature-based method, IDSV (Identification of Deleterious Synonymous Variants), for predicting harmful sSNVs in human genome by comparing deleterious sSNVs from the Database of Deleterious Synonymous Mutations (dbDSM) [6] with the putatively neutral sSNVs from VariSNP [18]. We computed an optimal set of 10 features selected from a wide variety of splicing, conservation, codon usage, sequence, RNA folding, translation efficiency, and function regions annotation features with the sequential backward selection method. The results on the benchmark datasets show that IDSV achieves a significantly improved overall performance based on the 10-fold cross-validation and independent dataset, and is capable of more accurately predicting deleterious sSNVs compared with other state-of-the-art methods.

## Methods

### Datasets

The positive (disease-causing) sSNVs were retrieved from the dbDSM (version 1.2), utilizing only the variants from ClinVar, PubMed database, or Web of Knowledge as disease-causing. This process results in a total of 300 deleterious sSNVs in the dbDSM database. To show the reliability of the prediction model, it is essential to employ a negative (neutral) data set. Here the negative data set was randomly extracted from VariSNP (version 2016-06-09) [18], which is a benchmark data set for benign variants. The final training dataset consists of 600 sSNVs (Additional file 1), where half are from the positive data set and the other half from the negative data set.

In order to further assess the performance of IDSV, we extracted an independent test set from the ClinVar database [19] (downloaded on December 14, 2017). In ClinVar, the values of clinical significance are based on the recommended rules by the American College of Medical Genetics and Genomics (ACMG) guidelines. In our study, the sSNVs labeled as ‘pathogenic’ or ‘likely pathogenic’ in ClinVar were considered as true positive sSNVs, and negative sSNVs were regarded as the ones with ‘benign’ or ‘likely benign’. Because bias may be introduced if negative sSNVs are found within genomic regions that differ substantially from regions containing positive sSNVs, such as those from different genes [11], we selected those putative negative sSNVs that are located in a gene with at least one positive sSNV. To ensure unique variants were used in the test set, we discarded variants presented in the training set. According to the above definitions, we obtained 153 deleterious sSNVs and 5178 benign sSNVs in 98 genes (Additional file 2).

### Feature representation

To construct a model that could best distinguish deleterious sSNVs from benign sSNVs, a total of 74 features are used to characterize potential deleterious synonymous variants, including conventional ones [10, 16, 20–22] and new ones [23, 24] exploited in this kind of study. To avoid having the discussion be too dense, these features are roughly divided into seven groups: splicing, conservation, codon usage, sequence, translation efficiency, RNA folding, and function regions annotation features.

Splicing features were described by 46 values, where 15 of them were obtained from SilVA [10], and the remaining 31 values were annotated by Skippy [20] and SPIDEX [16]. The conservation features were extracted by retrieving three tools, including SilVA (GERP++ score), SnpEff (100-way vertebrates conservation, Version 4.3) [21], and MyVariant.info high-performance web services [22]. Codon usage features (relative synonymous codon usage (RSCU) and variant-induced change of RSCU) were also obtained from SilVA. Sequence features were implemented with three tools, including SettleSeq Annotation 138 (http://snp.gs.washington.edu/SeattleSeqAnnotation138), MyVariant.info, and SilVA. The RNA folding features, which were obtained from SilVA, include the changes in the secondary structure folding energy and the diversity of the structural ensemble of pre-mRNA and mature mRNA, respectively. The translation efficiency feature was computed as the logarithm value of the adaptation index of the tRNA usage [23]. Functional regions annotation features were calculated based on the BED files of functional components from ENCODE consortium [24] and UCSC [25], including histone modifications, transcription factor binding site (TFBS), RNA binding proteins, open chromatin, all footprints, DNase I Hypersensitivity Clusters in 125 cell types and transcription factor binding site cluster track. In this study, we set all missing features to zero and normalized features with the z-score method. A detailed list of features and how they are derived can be found in Additional file 3.

### Feature selection

Feature selection is the process of selecting the effective features from the original set according to a suitable criterion. As an important step in designing classifiers, it could readily remove redundant features to improve the model performance. In this work, 74 multifaceted features were generated as described before. It is apparent that the models built based on these large sets of features would overfit the training data. Therefore, we used a wrapper-based feature selection algorithm based on sequential backward selection [26], in which features are sequentially removed from the original feature set until the removal of further features does not increase an objective function called criterion. Here, the criterion is AUC (the area under the receiver operating characteristic (ROC) curve) of 10-fold cross-validation on the training set.

### Model construction

The classification model for predicting deleterious sSNVs was based on RF, which is an effective supervised method that demonstrates high prediction accuracy whilst efficiently avoiding the overfitting problem. In this study, the randomForest R package (version 4.6–12) was employed and executed with 10-fold cross-validation and the independent test set. In order to achieve good experimental results, two main parameters, the number of trees to grow for each forest (*ntree*), and the number of input variables randomly chosen at each split (*mtry*) of the RF were optimized using a grid search method in the range of *ntree ϵ* {50, 1000, *by* = 50} and *mtry ϵ* {1, 10, *by* = 1} based on the results of 10-fold cross-validation on the training set.

### Performance evaluation

Predicting an sSNV as deleterious or benign is a binary classification problem, and many measures have been introduced for validation issues. Besides the AUC mentioned above, the prediction performances were also evaluated by the Recall, Precision, and F-measure [27]. These evaluation measures are defined as:

$$ Recall=\frac{TP}{TP+ FN} $$

$$ Precision=\frac{TP}{TP+ FP} $$

$$ F- measure=\frac{\left({\beta}^2+1\right)\times Precision\times Recall}{\beta^2\times Precision+ Recall} $$

where *TP*, *FP*, *TN*, and *FN*, and *β* correspond to the number of true positive samples (correctly predicted deleterious sSNVs), the number of false positive samples (benign sSNVs incorrectly predicted as deleterious sSNVs), the number of true negative samples (correctly predicted benign sSNVs), the number of false negative samples (deleterious sSNVs incorrectly predicted as benign sSNVs), and the relative preference of Recall against Precision (set as the ratio of the majority class size to the minority class size to emphasize the Recall), respectively.

## Results

### Selection of optimal features

The main goal of this study is to build effective and accurate model to predict deleterious sSNVs. To this end, identification of a set of informative features is critical for performance boosting and subsequently can enhance our understanding in the molecular basis of deleterious sSNVs. Based on the method of wrapper-based feature selection algorithm using sequential backward selection mentioned in Feature selection section, a set of 10 optimal features were obtained from 74 features, which included splicing (6 features), conservation (1 feature), translation efficiency (1 feature), sequence (1 feature), and function regions annotation (1 feature) and listed in Table 1. We found that splicing features dominate the top-10 list, suggesting that splicing features are more predictive than other properties in determining deleterious sSNVs.

**Table 1:** sSNVs were annotated with a set of 10 optimized features spanning five distinct classes of infomration relevant to assessing the harmfulness of sSNVs

Feature name	Description	Type
Sequence feature		
DSP	Mutation site distance to the nearest splice site	Integer
Function regions annotation		
TFBS	Whether the variant is in transcription factor binding site?	Bool
Splicing		
MDE	Minimum distance as a proportion of half the exon	Numeric
DVE	Distance of the variant across the exon as a proportion	Numeric
ese-dens	Density of neighborhood inference-exonic splicing enhancers hexamers in the exon sequence	Numeric
MES	Max splice site score	Numeric
SR-	SR-protein motifs lost	Numeric
dPSIZ	The z-score of dPSI (the predicted change in percent-inclusion due to the variant, reported as the maximum across tissues) relative to the distribution of dPSI that are due to common variant	Numeric
Conservation		
verPhyloP	Vertebrate PhyloP at the mutation position at mutation position	Numeric
Translation efficiency		
TE	The tRNA adaptation index of the tRNA usage	Numeric

**Fig. 1:**
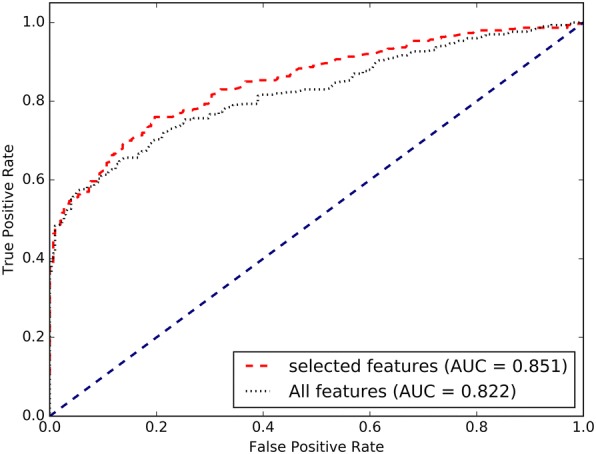
The ROC curves of prediction methods with and without feature selection using the 10-fold cross-validation on the training set

To quantitatively assess the performance of feature selection algorithm in our method, we compared it with the candidate full feature set. Figure 1 shows the ROC plots of RF model performance with the selected features based on feature selection algorithm and the 10 selected features of 10-fold cross-validation. As can be seen from Fig. 1, the AUC of the selected features model with *ntree* = 500 and *mtry* = 3 are about 3% higher than the full feature set model, which demonstrate that the feature selection algorithm is able to achieve better performance with less computational cost and reduce the risk of overfitting.

To further understand the contributions of the selected features, we also compared our method’s 10-fold cross-validation performance removing each feature from the analysis. As can be seen from Table 2, compared with the models constructed by leaving out any features, the model with all selected features has the highest Recall (0.700) and the AUC (0.851). Removing features related to sequence and function regions annotation does not substantially affect performance. However, removing either conservation or new translation feature causes model’s performance to drop substantially. Although splicing features is the dominant factor for diseasing-causing sSNVs, not all the splicing features have the same potential for performance improvement, such as SR- and dPSIZ. While some features are weakly informative, they may have a good complementarity and thus collectively contribute to the harmfulness prediction.

**Table 2:** Prediction results by subtracting each feature using the 10-fold cross-validation on the training set

Feature	Recall	Precision	F-measure (β = 1)	AUC
All features	**0.700**	0.820	0.755	**0.851**
No SR-	0.697	0.823	0.755	**0.851**
No MES	0.667	0.810	0.731	0.829
No MDE	0.677	0.820	0.742	0.834
No DVE	0.697	0.816	0.752	0.846
No ese-dens	0.693	0.835	**0.757**	0.845
No dPSIZ	**0.700**	0.813	0.752	0.850
No verPhyloP	0.697	0.768	0.731	0.829
No TFBS	0.690	0.818	0.749	**0.851**
No DSP	0.683	**0.837**	0.752	0.845
No TE	0.673	0.798	0.731	0.829

The highest values are highlighted in bold

### Comparison of different machine learning classifiers

**Fig. 2:**
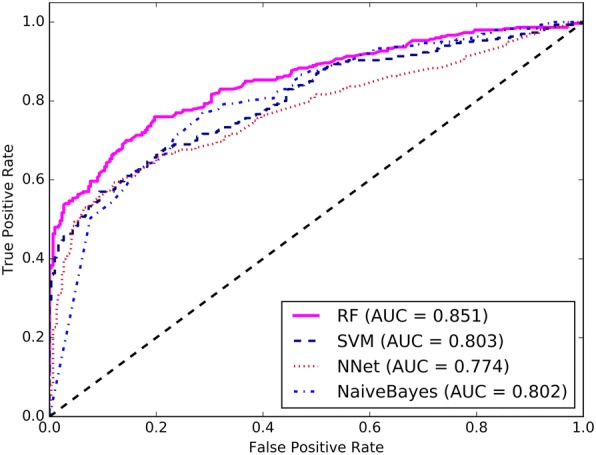
The ROC curves of different machine learning methods using the 10-fold cross-validation on the training set

In order to identify the best suitable machine learning technique for predicting deleterious sSNVs, we comprehensively evaluated the performances of SVM, Neural network (NNet), Naive Bayes, and RF based on the selected 10 features. All these algorithms were implemented using the R package with the parameters optimized. The performance comparison of different machine learning classifiers with 10-fold cross-validation and the independent test set is listed in Fig. 2 and Additional file 4. It can be seen that RF outperformed SVM, NNet, and Naive Bayes with the AUC increased by more than 0.048, 0.077, and 0.049 respectively based on the training set. When evaluated on the independent test set, the AUC of RF was also higher than those of SVM, NNet, and Naive Bayes, with ΔAUC of 0.021, 0.072, and 0.023, respectively. All the above findings indicated that RF gives the best predictive performance compared with SVM, NNet, and Naive Bayes.

### Comparison with other methods

In this section, we compared the performance of IDSV with other methods. Table 3 summarizes the performance comparison of different methods on the same independent test set. Among these approaches, SilVA, DDIG-SN, and TraP are synonymous-specific methods, while FATHMM-MKL and CADD are general approaches for all types of single nucleotide variants. We have not compared IDSV with regSNPs-splicing, as too many sSNVs’ prediction scores are not available from its web server. We also found that CADD yielded no prediction for one variants. Another method, DDIG-SN, have 48 missing predictions. We decided to retain these variants in our test set after checking that the evaluation results of the compared methods were not affected.

**Table 3:** Performance comparison of different methods on the independent test set

Method	Recall	Precision	F-measure (β = 34)	AUC
IDSV	**0.765**	0.098	**0.761**	**0.869**
CADD	0.320	0.081	0.319	0.700
FATHMM-MKL	0.712	0.053	0.704	0.751
SilVA	0.490	0.581	0.490	0.844
DDIG-SN	0.298	**0.789**	0.298	0.854
TraP	0.575	0.518	0.575	0.827

The highest values are highlighted in bold

Overall, we can see that IDSV showed high success rates in contrast to the other methods. Our method IDSV can correctly predict deleterious sSNVs from the data set with recall = 0.765 and precision = 0.098, which means that our method can correctly predict 76.5% of the true deleterious sSNVs, and 9.8% of the predicted deleterious sSNVs are identified as true deleterious sSNVs. We can see that the precision of our method was lower than for three synonymous-specific methods, SilVA, DDIG-SN, and TraP. Although our method achieves the high recall at the expense of some precision, the AUC score indicates that an adequate balance is still achieved (The AUC score for IDSV is 0.869, while the other methods have AUC scores in the range of 0.700–0.854). The two general approaches, FATHMM-MKL and CADD, have low recognition accuracy in predicting deleterious sSNVs. A possible reason is that these approaches a designed to discriminate whether a single nucleotide variants is harmful or not, not optimized for predicting deleterious sSNVs.

Beside AUC score, another important alternative metric, F-measure, was also used for performance assessment of model, as the independent test set was highly imbalanced with more benign than disease-causing variants per gene (the overall ratio of 34:1, so the coefficient ***β*** of the F-measure was set to 34). The F-measure of IDSV is 0.761, which represents a relative improvement of 5.7% over the second best method, FATHMM-MKL, which yielded the F-measure of 0.704.

Due to the independent test set was highly imbalanced, we were interested in whether IDSV could retain its performance when tested on a fully balanced data set. In this test, we randomly extracted 153 benign sSNVs from the full negative independent test set. The final balanced data set consists of 306 sSNVs (153 deleterious and 153 benign sSNVs). We aggregated the results across five runs, each time with a new random subset of benign sSNVs. As shown in Table 4, IDSV is able to retain most of its prediction performance with the AUC of 0.868 and F-measure of 0.722. We also calculated *P*-values with the two-tailed, paired *t*-test [28] to compare the performances of IDSV and other methods. It can be seen that the P-values for the difference between IDSV and other approaches are much smaller than 0.05, which suggest that IDSV has a significant advantage over the pioneer approaches in predicting deleterious sSNVs.

**Table 4:** Performance comparison of different methods based on the balanced subset of the independent test set in which benign variants were randomly selected from the full negative independent test set. We repeated this process 5 times with different random subsets of benign variants and averaged the results

Method	Recall	Precision	F-measure (β = 1)	AUC	*P*-value
IDSV	**0.765 ± 0.000**	0.781 ± 0.022	**0.772 ± 0.011**	**0.868 ± 0.008**	*
CADD	0.320 ± 0.000	0.760 ± 0.041	0.450 ± 0.007	0.698 ± 0.018	9.452e-07
FATHMM-MKL	0.712 ± 0.000	0.660 ± 0.026	0.685 ± 0.014	0.753 ± 0.019	0.0007962
SilVA	0.490 ± 0.000	0.977 ± 0.017	0.653 ± 0.004	0.844 ± 0.017	3.211e-05
DDIG-SN	0.298 ± 0.000	**0.996 ± 0.010**	0.459 ± 0.001	0.853 ± 0.006	5.957e-07
TraP	0.575 ± 0.000	0.971 ± 0.012	0.723 ± 0.003	0.848 ± 0.043	0.001015

The highest values are highlighted in bold. *Denotes the reference when calculating the P-value

### Additional tests on the training set of our method

For a machine learning problem like the one we are tackling, different settings in training set may cause overestimate or underestimate of the actual performance. Here we carry our two additional tests under different training settings to demonstrate the robustness of IDSV.

Currently, the set of benign sSNVs are chosen from VariSNP. To investigate whether different sources of benign sSNVs in the training set affect the prediction performance, we randomly chose 300 benign sSNVs from ClinVar as well. Using the new set of benign sSNVs, we retrained the model and obtained the predicted AUC scores for the 10-fold cross-validation on the training set and the independent test set. The results for the 10-fold cross-validation on the training set and the independent test set changes from 0.851 to 0.862, and 0.869 to 0.874, respectively, which indicate that the AUC scores are similar either using VariSNP or ClinVar to form the set of benign sSNVs in the construction of prediction model.

Because there are much more benign sSNVs than deleterious sSNVs, it is an interesting question to test whether different sizes of benign sSNVs in the training set affect the prediction performance. In the Method section described earlier, we choose the size of the benign sSNVs to be the same as the deleterious sSNVs in the training set. Here we investigate whether increasing the size of the benign set to 10, 20, 30, 40, and 50 times of the deleterious set has any effect on the predictive performance. The predicted AUC scores are shown in Additional file 5. We can see that, overall, increasing the size of the benign sSNVs in the training data set has little effect on the prediction performance using 10-fold cross-validation and independent test set, which suggests that the performance of IDSV does not change much with different level of deleterious/benign imbalance.

## Discussion

In this work, we described a feature-based computational IDSV for identifying deleterious synonymous variants. Both the new feature based on the translation efficiency and function regions annotation traditional features based on splicing and conservation are used as the input to RF classifier. Our analysis implies that besides splicing and conservation features, the new translation efficiency feature is also an informative feature for identifying deleterious sSNVs. While the function regions annotation and sequence features are weakly informative, they may have the ability to discriminate deleterious sSNVs from benign ones when combined with other features. The performance of our IDSV was firstly evaluated using the 10-fold cross-validation and further validated using an independent test set. The experimental results show that IDSV can provide favourable or at least comparable performance compared with all the previous methods.

## Conclusions

In conclusion, IDSV is an efficient feature-based prediction approach for deleterious sSNVs by using a wide variety of features. We believe that IDSV can be a useful model to facilitate exploration of deleterious sSNVs with the increasing availability of sSNVs data from the next-generation sequencing technologies. The data and source code are available on website http://bioinfo.ahu.edu.cn:8080/IDSV.

## Additional files

Additional file 1: Deleterious and benign sSNVs used in the training set. (XLS 180 kb)

Additional file 2: Deleterious and benign sSNVs used in the independent test set. (XLS 1411 kb)

Additional file 3: List of all 74 features used for feature selection and model construction. (XLS 47 kb)

Additional file 4: The ROC curves of different machine learning methods on the independent test set. (TIF 416 kb)

Additional file 5: Summary of AUC scores based on 10-fold cross-validation and the independent test set when benign variants are 10, 20, 30, 40, and 50 times of deleterious variants in the training set, respectively. (XLS 19 kb)

## Abbreviations

ACMG: American College of Medical Genetics and Genomics
AUC: The area under the ROC curve
dbDSM: Database of Deleterious Synonymous Mutations
DSP: Distance of the nearest splice site
RF: Random forest
ROC: Receiver operating characteristic
SNVs: Single nucleotide variants
SPANR: Splicing-based Analysis of Variants
sSNVs: Synonymous single nucleotide variants
SVM: Support vector machine
TFBS: Transcription factor binding site
TraP: Transcript-inferred Pathogenicity
