# Supplementary figures and images for: Computational identification of deleterious synonymous variants in human genomes using a feature-based approach
Source: BMC Med Genomics. 2019 Jan 31;12(Suppl 1):12. doi: 10.1186/s12920-018-0455-6 (PMC6357349; doi:10.1186/s12920-018-0455-6)

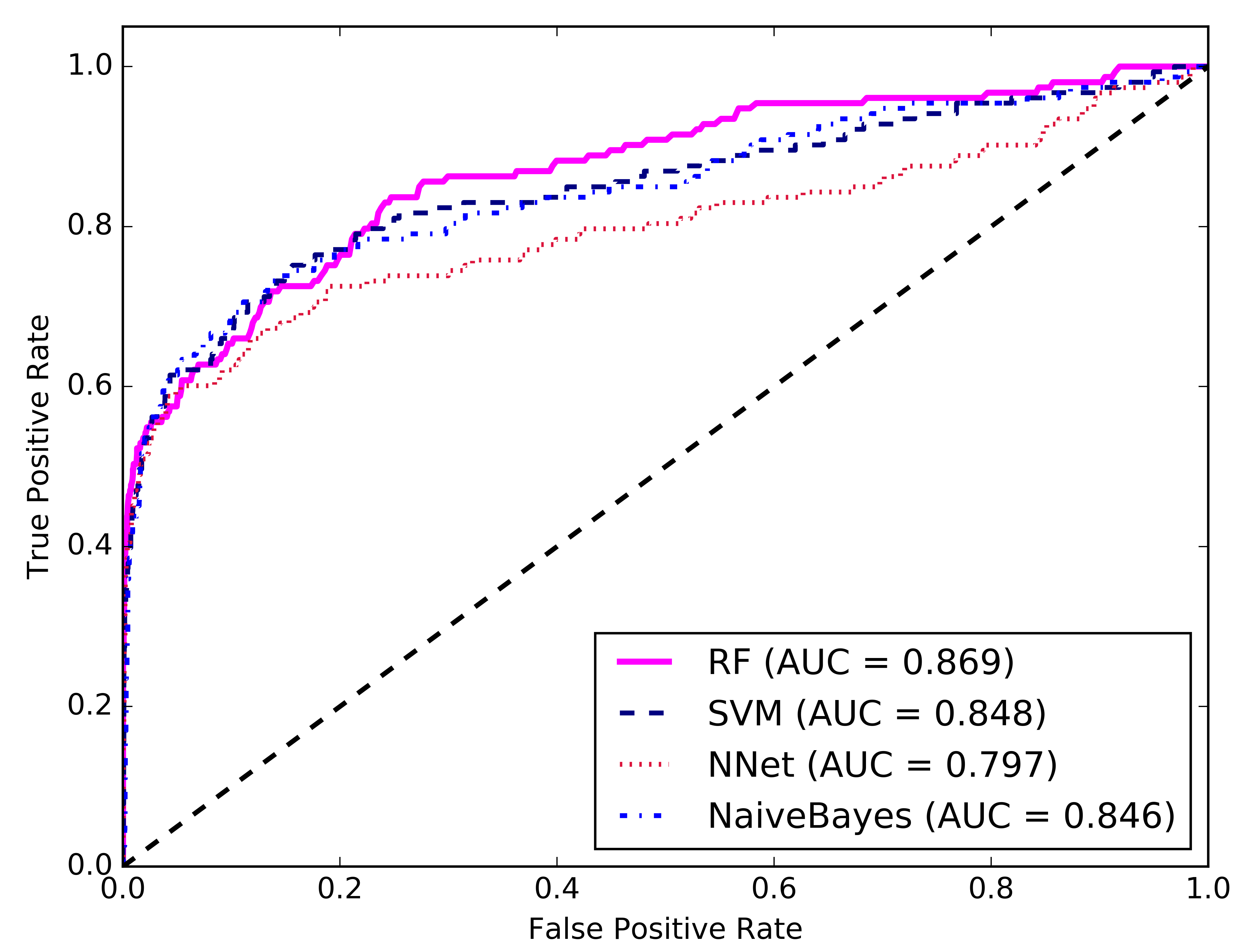

Supplement: Supplementary file 4 — The ROC curves of different machine learning methods on the independent test set. (TIF 416 kb) [file 12920_2018_455_MOESM4_ESM.tif]
